# Reviews and Records in Anatomy and Physiology

**Published:** 1854-07

**Authors:** Waldo I. Burnett

**Affiliations:** Boston


Reviews and Records in Anatomy and Physiology.—By Waldo I. Burnett, M. D., Boston.—1. *Psorospermia.** Lieberkühn has furnished a communication of considerable length and detail on this subject, to which we refer particularly from its interesting relations. As a key to the article we may give his conclusions, as follows:—

"The kidneys of some frogs contain cysts which enclose contents of very manifold characters, namely:—1. A peculiar, uniformly divided, granular mass. 2. This mass encompassed by small groups of an oval or fusiform shape. 3. Fusiform bodies, invested with a structureless memberane. 4. Develöped psorospermatic corpuscles. These different objects are found wholly or partially in one and the same cyst. The mature psorospermial corpuscles usually contain three to five baton-like or ellipsoid or globular diaphanous corpuscles which are structureless; they usually have also a rather large nucleus. The diaphanous corpuscles are seen moving and springing in their capsules, and the nucleus is thereby moved about hither and thither. Such kidneys contain also free amœba like corpuscles, and gregarina-like bodies largely nucleated."

These formations are by no means common,—our author having found them in four cases only out of a thousand specimens he has examined.

But in other animals, and in other organs he has found similar formations, as have others, such as Muller, Gluge, Leydig, before him. In fact, these psorospermial forms occur in both a free and a cystic state in different tissues.

The question is certainly a most interesting one: What signification shall be put upon these singular animal-like forms? But this only one passage in the comprehensive question, What are most infusorial forms not evidently of a vegetable nature which every microscopist meets with perhaps daily in his studies? The subject certainly is not yet ripe for decision, but we may allude to it in a suggestive point of view. Both Kauffmann and Lieberkuhn, in watching these Psorospermia in glasses on their tables,

* *N. Lieberkühn.* Ueber die Psorospermien, in *Müllers* Arch, 1854, Hft. 1, p. 1–25.

have observed that they multiply by a segmentation of their nucleus, and that the product of this division resembles precisely the parent. In some specimens observed by Lieberkuhn, taken from a dog, he found the parent vesicle to contain sometimes 16 segmented globules. But here the observations unfortunately ceased, and we are not aware that in any case or instance they have been extended beyond this point; that is, so as to show that the offspring of this segmentation which so closely resembles its parent pursues the same course and produces by segmentation a third series. The doctrine that individual animal forms may be unicellular or that an animal may be composed solely of a single cell, as advanced by Siebold and Kölliker,* we regard as wholly untenable in the present state of science; for, aside from its being against the general analogy of individual zoological forms, it has not yet facts enough to sustain it merely as a point of observation. The cell is indeed typically the primordium of all organized forms, but true individual animal life seems to involve a cycle of relations not implied in single cells; in other words, these last must always lose their character as such in a definite form which belongs to the individual. Extended researches in Microscopy in all directions of the organic world,—all tend to establish the doctrine that sex lies behind all true individual forms,—that the ovum is the point of departure on one side, and the spermatic particle on the other,—and that by the conjunction of the two a new individuality is produced. There is indeed a most striking and beautiful uniformity between the simple cell and the ovum in a morphological point of view, or between the cell and the parent sperm-vesicle, thereby indicating a unity of idea and place in the first expression of life and the functional means of its cycle of actions; but without wishing to be mystical, it appears to us that life as expressed under the individual whether in its first or last forms,—as an egg ready to develop, or as a complete animal, rises high above and implies a great deal more than simple cell-conditions. We argue, then, that all true animals arise primarily or secondarily from ova, and therefore have sex, and that those animal-like forms so often seen as parasites or entozoa in animals, must for the present stand undetermined, or if any interpretation is to be put upon them, they may be regarded as undeveloped forms of true animals, passing through various metamorphotic changes to which we have some clew in the remarkable phenomena of *alteration of generations.*

If the contents of the lower portion of the intestine of a frog be examined under the miscroscope, there will usually be found innumerable moving particles which give a very life-like aspect to the whole field. These belong to the infusorial genus *Bodo* of

* *Siebold* and *Kölliker*. See their Zeitsch. für wissenschaftliche Zoologie, I, p. 270, and ibid p. 1.

Ehrenberg. Examined with the highest and best microscopic powers, they are found to be composed of a more or less globular head, to which is attached a thread-like tail of considerable length. This head taken by itself has all the appearances of a simple cell; it is uncleated and even nucleolated; yet the whole body moves about by means of its tail in a most animal-like manner, and in studying the field one can hardly divest the mind from the opinion that they are true animals.

The intestinal canal of many insects likewise, especially those feeding in damp, moist places, will often be found teeming with forms so large and numerous that it is singular that the insect should live. Many of these forms are composed of a more or less globular sac filled with punctiform matter in which lies a round nucleus; at one extremity of this sac is an orifice surrounded by a circle of cilia. Others are more vermiform, regularly wrinkled, but apparently non-uncleated. These belong to the Gregarinæ, and are the forms upon which Siebold and Kölliker have based their doctrine of unicellular animals. Other instances might be cited, equally prominent, which almost daily come under the eye of the microscopist in his studies in the lower department of the animal kingdom.

Our own observations upon these objects have not led us to the view that they are, any of them, perfect individual forms;* on the other hand, research is constantly reducing their generic numbers, on the other side, by showing that many so-called genera are only different developments of one and the same form; and by removing them, on the other, from that *Receptaculum omnium animalium et plantarum*—the Infusori—it being shown that they are only germs or larval forms of some of the inferior classes.† The recent investigations of Siebold and others, in Helminthology, to which we shall soon allude, have shown how varied may be the larval forms, how dissimilar from the true adult animal, and how remarkable the localities in which they occur, in the case of many of the Entozoa. In the case of the Psorospermia, or the Gregarinæ, or other forms which have been grouped under special genera or species, we must wait further research, and they will then, we think, be shown to be undeveloped forms. As to the question of the animal or vegetable nature of such organism, it seems equally obscure, for the older criteria by which animals were separated from plants have long since been regarded as invalid; and some of those which in late years have been regarded as among the most constant, have quite recently been declared unsound.

Robin has argued that the Psorospermia are plants because they

---

* For *Bodo* see Boston Journal of Natural History, vi, No. 3, 1853, p. 318.

† Thus *Agassiz* has shown that *Paramœcium* and *Bursaria*, &c., are only laval forms of Planariæ. See Annals of Nat. Hist., vi, 1850, p. 156. *Euglena*, *Amœba* and others, will most probably meet with a like resolution.

contain cellulose; but as the investigations of Kölliker, and of Löwig and Schacht have shown that this substance occurs indubitably in animal tissues, this can no longer be considered as a criterion.* We are reduced to *voluntary motion* as the only now known differential criterion on this subject, and as this must be a point on which different observers will vary, the subject must still remain unsettled; but we protest against any fusion of the two organic kingdoms on this obscure arena.

2. *On the Mermithes.*† A memoir of great worth has recently appeared upon these singular parasites, which has a double importance of quite clearing up the history of these animals in all their stages, and of furnishing a contribution to the histology of the lower animals of a most valuable character. This memoir is by G. Meissner of Munchen, under the direction of Siebold, who furnished him with specimens and other opportunities for its successful prosecution. Seldom have we met with a paper of more careful and extended detail, and which leaves so little behind for investigators in the same direction. Added to this textual detail, each and every anatomical point is illustrated by admirably executed figures. With our limited space we can at best notice only a few of the more prominent points of this paper.

In the first place it should be remarked that the natural history of the Gordiacei was for a long time quite obscure and little understood, and many detached observations not of a parallel character did not improve the subject. To the sagacity of Siebold we are indebted for the successful resolution of the whole enigma, and the results he has obtained are as singular as new.‡ It appears that these animals live part of their life as a regular entozoon, and the rest as an independent being. And what is most remarkable, they enter the animals in which they are for a time parasites, not in the form of eggs, as do other Helminths, but as more or less developed forms. The animals in which they live as parasites are almost exclusively insects of different orders in both the larva and imago state. In the abdominal cavity of the larvæ of *Yponomeuta albicans*, Siebold found numerous undeveloped forms of *Mermis albicans.* Watching them he found that after further growth, they perforated the skin of these larvæ and made their escape. These freshly-escaped individuals were all sexless, but contained each a considerable *corpus adiposum*, at the

---

* For full reference to the subject of cellulose in the tissues of the lower animals, see *Siebold* and *Stannius's* Comparative Anatomy, Amer. ed. i, § 172.

† Beiträge zur Anatomie und Physiologie von Mermis albicans. Von Dr. *Georg Meissner*. In *Siebold* and *Kölliker's* Zeitschrift für wissenschaftliche Zoologie, v, 1853, p. 207–285.

‡ *Siebold*. See the Entomol, Zeitung zu Stettin, 1848, p. 292, 1850, p. 329; also, Beiträge zur Naturgeschichte der Mermithen, in *Siebold* and *Kölliker's* Zeitsch. für wissenschaftliche Zoologie, v, 1853, p. 211.

expense of which their sexual parts were subsequently developed. These animals crawled about and soon entered some damp earth, where they remained several months, during which time they were further developed, changed their skin, copulated and laid their eggs. The embryos hatched from these eggs had the filamentoid form of the adults, and as Siebold conjectured that they intended to come to the surface for the sake of entering in their turn young insects, he procured quite young larvæ of this same insect and put them in an hour-glass together with the young Mermithes. In a few hours they had entered the body of these larvæ, two or three in each. Siebold had the precaution to make this point certain by carefully examining these larvæ previously and determining that their bodies were free of these parasites. After this, the same round of life is again passed. It would appear, then, that these animals pass their earlier (but not their embyronic) conditions of life whereby they attain their development—in fact a proper larval state—in the bodies of insects, and that their life as distinct sexed individuals is free and non-parasitic. Siebold found this species in very many genera of Lepidoptera; also, in different species of Orthoptera, Coleoptera and Diptera. We may mention that the common cricket as also some other Orthoptera, are frequent recipients of *Mermis*, and we have seen many specimens of this kind. Until Siebold's recent contributions we had supposed, in common with other naturalists, that these Helminths merely hibernated in these insects, but this is now quite improbable.

So much for a brief reference to the mode of life of these animals: we will now turn and glance at some of the important histological points as wrought out by Meissner.

*Cutaneous System.*—Omitting the very full details given of the structure of the skin in these animals, its composition of three distinct layers, &c., we will allude only to the fact that *Chitine* enters likewise into its formation. This fact is important as corroborative of other observations. Chitine was formerly supposed to belong exclusively to the teguments of the Arthropoda, being particularly prominent in the skin of insects; but recent chemical analyses of the teguments of lower animals show that it occurs in nearly every class of the Invertebrata.* It can therefore no longer be regarded as having diagnostic characteristics for certain classes, but sustains relations to the external dermic skeleton of the Invertebrata generally, analogous to those of bone in the four classes of Vertebrata.

---

* Besides the present case we would refer to the following: *Grube, Müller's* Arch. 1848, p. 461, and *Wiegmann's* Arch. 1850; *Schultze*, Beit. zur Naturgesch. d. Turbellarien, p. 33, and *Leuckart* in *Siebold* and *Kölliker's* Zeitsch., 1851, p. 192, and *Wiegmann's* Arch. 1852, p. 22.

*Muscular System.*—This was found quite developed, and it is a singular fact that all the muscles have a longitudinal direction. Transverse muscles do not exist. But Meissner has indicated a histological feature of muscular tissue in these animals, which deserves notice. It is well known that straited muscular fibre is rather limited in its distribution among the Invertebrata. As stated in another place,* we have not observed it below the Articulata, and have regarded it as actually absent in the remaining classes—the Cephalopoda, Cephalopora, Acephala, Annelides, Turbellaria, Helminthes, Echinodermata, Acalephæ and Polypi. Now, we have hitherto supposed from observation that the fibre being the true embryological element of muscle, a further division into the fibrillæ occured only in the higher form of this fibre, the so-called *striated* muscle; in other words, that a fibrillated structure of muscular fibre was found only in the striated form. But Meissner describes the fibre of *Mermis* as readily capable of being split up into longitudinal fibriæ of the most regular and delicate character, and yet neither these fibres nor fibrillæ are properly transversely striated. He remarks, however, that an appearance like striation is sometimes observed by a wave-like contraction of the fibre.† Results of this character which the more careful research of the present day is developing, in studies of the lower animals especially, fully indicate that the subject of muscular tissue is not well understood as to its manifold variations of form; at least, after we have left the typical forms of the higher animals. Thus, as company for the present instance, I may mention that Leydig‡ found the mucles of the alimentary canal of *Artemia* among the Crustacea, composed of spindle-shape instead of disc-like elements, so arranged, points and bases alternating, as to form a symmetrical fibrilla. In conclusion upon this system, we may remark that Meissner found here no sarcolemma, and no perimysium of the muscular layer.

*Nervous System.*—The researches of this investigator in this direction have particular interest, because this system has been generally denied to the Gordiacei, and if seen by previous observers it was only most unsatisfactory.§ But the histology of this system is quite as quite as interesting.

---

* Am. Jour. of Science and Art, Jan. No.. 1854, p. 89.

† We suspect it is the same wave-like aspect that has been often mistaken for striation in the muscles of some of the lowest animals, thereby leading to no little discrepancy among observers in their statements. See this Journal, Jan. No., 1854, p. 92, notes.

‡ *Leydig.* Ueber Artemia salina und Branchipus stagnalis, in *Siebold* and *Kölliker's* Zeitsch., iii, p. 280, Taf. viii, fig. 6.

§ *Berthold* and *Blanchard* both supposed they saw cords which might be nerves, but their observations were wholly unsatisfactory;—for references see *Siebold* and *Stannius'* Comp. Anat. Amer. ed., vol. i, § 104, note 5.

Meissner found it so developed that he divides it into three portions:—a central, a peripheric and a splanchnic portion.

The *central portion* is divided into two parts, one at the cephalic, the other at the caudal extremity of the body. In the first, are two anterior and two posterior cephalic ganglia, and an œsophageal ring composed of a superior and an inferior ganglian united by lateral commissures. In the second part, situated in the tail, there are three fusiform ganglia, of like character but smaller than those of the head.

The *peripheric portion* consists of six filaments given off from the upper part of the anterior cephalic ganglia which go to as many papillæ on the head and probably organs of sense,—of two lateral cords arising from the superior œsophageal ganglion, which traverse the sides of the body, giving off filaments to the muscles, the skin, &c., and of some smaller twigs from the cephalic centers for the muscles of that region.

The *splanchnic portion* consists of two latteral trunks arising from the œsophageal ganglion, which soon meet and unite on the median line of the body, forming one cord which extends to the tail. From this cord are given off filaments to the organs of vegetative and reproductive life.

The three cords thus formed, having traversed the body, end each in one of the three ganglia above described. We can here allude to only one more point in the disposition of the nervous system; this is the final termination of the nerve-filament in muscle. According to our author, a twig enters the muscular fibre at right angles to the course of the latter, and upon its entrance divides into two twiglets, one of which runs with the fibre one way and the other the opposite, and is lost in the muscular tissue.†

The histology of this system in so minute animals as these, worked out by an observer so expert and faithful as Meissner, presents many note-worthy points.

The ganglia in question are composed exclusively of ganglion-cells or globules which appear to be the infundibuliform expansion of as many nerve fibres that compose the nervous cord connecting these ganglia with the general system. There are none of the so-called nerve cells usually found in nervous centres—in fact these central masses rather resemble true ganglionic formations, excepting that they are terminal instead of on the course of a nervous cord. The description and figures, especially the latter, of Meissner are so good, as to leave no doubt that there is here a direct continuity of the nerve-fibre with the ganglionic vesicle.

---

† As *Meissuer* observes, a similar disposition is mentioned by *Doyère* (Ann. d. Sci. Nat., 1840, xiv, p. 346) in the muscles of the Tardigradi, and by *Quatrefages* (ibid, 1843, xix, p. 300) in the Eolidina, some Annelides and Rotatoria.

In a former review* we alluded to some discrepancy on this point, and as this continuity had been observed by some, and yet not seen by others who had searched carefully for it, we suggested that this direct connection, when present, might be an exceptional condition. But numerous researches since published, and especially the very complete memoir of Axmann,† represent this as a very common form of disposition of the elements of nervous centres in man and the mammalia. The subject is indeed somewhat obscure in a functional point of view, for what is the interpretation of this direct continuity of the vesicular with the tubular portion of this system? Certainly it is not the essential condition of function between the two, or all nerve fibres would terminate in this manner and there would be no ganglionic vesicles but those having this connection. But this, as is well known, is far from being the case. We leave the subject until another time. As to the structure of the peripheric nerves, our author describes them as having at first a distinct fibrillated structure as usual, but that this last gradually disappears and the nerve appears as a homogeneous cord. But from our own investigations upon the terminal nerves of some insects, we suspect that this disappearance of the true fibrillæ may have been apparent and not real; for we have, in the cases referred to, thought that the like was true, but using higher powers with some reagents, the fibrillæ were seen. We think therefore that whatever may be made of termination of the nerve-fibre, the fibrilla structure is never lost.

*Digestive Apparatus.*—This structure, according to Meissner, presents so many peculiarities and is so widely different from any thing observed in other animals, that we almost relinquish any attempt to give even a brief description of it, without the aid of figures. In the first place, the alimentary canal has no anal or excretory passages, and therefore the food and assimilation must be such as to leave little or no so-called fæcal matter.

From the circular buccal orifice proceeds a semi-canal a short distance, when it passes into another structure. This semi-canal is the œsophagus. The structure into which it passes is a tube quite small at first but which soon expands and is filled with a finely granular spongy-like substance, and is alternately dilated from side to side into sacs. Through this laterally varicose tube the semi-canal of an œsophagus extends to its very end. Suppose then a tube with alternate lateral dilatations, filled with a spongy substance, and through which runs a semi-canal or half-tube like an œsophageal groove. Each of these dilations has an inversion—a folding in of its internal membrane, producing an unfundibuliform body in the dilatation itself. This body opens

* Am. Jour. of Science and Art, Sept. No., 1853, p. 253.

† *Axmann*, Beitr. z. mikroskop. Anat. u. Phys. d. Ganglien-Nervensystems des Menschen u. d. Wirbelthiere. Berlin, 1853.

through a prolongation of the external membrane of the dilatation, which is continuous into a tube connecting with some adipose receptacles.

To perhaps make the matter more clear, fancy the human alimentary canal without an anal opening, with alternate stomachs throughout its course, filled with a semi solid granular substance; and that directly through it ran a half-tube; and that each stomach had a folding in of its internal lining forming a globular body, the neck of which passed off at right angles by a continuation of the peritoneum, into a tube which connected with receptacles of nutrition;—this would convey some idea of the most singular structure of the digestive canal of these animals.

The food passes along the semi-canal or groove, is gradually absorbed by the spongy substance filling the dilatation, thence passes into the invested body by endosmotic absorption and is then conveyed as assimilated material into the fat-receptacles which lie in the cavity of the body. These receptacle are storehouses of nutriment and are particularly enlarged and developed during the larval condition,—their contents being used for the formation of the sexual parts afterwards. Now as there is no vascular system in these animals, the dispersion of the nutrient material for the growth and substance of the various tissues must occur by permeation and endosmosis from the fat-bodies which extend over and between all the organs. This assimilation without any particular excretion is a remarkable fact; but it appears more conceivable when we bear in mind the economy of the animal. Its larval or parasitic state is like that of insects—merely preparatory for the ulterior changes of its full development. During this time its food is probably mostly pure fat which has only to be taken up and stowed away for material of the development of the reproductive organs. This last ensues during a quiescent state, and after the full discharge of the sexual functions, the animals probably die.

*Genitalia—Males.*—The disparity in numbers of males and females was remarkably wide—our author having found only three males among several hundred specimens examined. He divides the internal organs into *testis*, *vas deferens*, *vesicula seminalis*, and *ductus ejaculatorius;* but these are all continuous, forming a cæcal tube stretching from the anterior portion of the body to the caudal extremity. The testis consists of the infundibuliform cæcal extremity of this tube and is lined with nucleated, epithelial (?) cells.

The external organs consist of two penises situated one on each side of the *Ductus ejaculatorius* in a sheath. They are composed of two somewhat curved half-canals disconnected when unprotruded with the internal organs; but when protracted, they form a more or less closed tube projecting beyond the external orifice of the duct.

*Females.*—Meissner divides the internal female organs which are double, into five portions: *ovary*, *vitellus-organ*, *albumensac*, *tuba*, and *uterus.* Their names indicate their respective functions and we can here enter into no description of their intimate structure.

In this connection should be noticed one point not a little remarkable, that is, a kind of hermaphroditism occurring in these animals.

Meissner found individuals which had perfectly well-formed internal female genital organs, but whose caudal extremity was wholly male. Thus, there were the penises, with their protractor and retractor muscles, their sheaths—in fact, all the external organs of the male, yet in these individuals no trace of internal male or of external female organs could be found. Moreover these organs present precisely the same characteristics as though in proper males and females, and had also a functional activity,—eggs being found in the ovaries, &c. But this anomoly was not ever found in the inverse sense, that is female external and male internal organs. Here then is presented the striking peculiarity of an animal having double systematically developed internal organs of one sex, and at the same time perfectly formed external organs of the other sex. This hermaphroditism, it will be seen, is like that of other animals only in name; for in these last the double sex is at the expense of the symmetry, one side being female and the other male, or it is due to modifications of analogous facts by different grades of development, thereby destroying generally the functional perfection and completeness of each or one of the forms of the sexual organs. But here we have a perfectly symmetrical female internally, with an equally symmetrical male externally, with no fusion of parts.

In regard to the development of the spermatic particles, our author's researches have been minute and quite complete. His results confirm the doctrines of Kölliker, Wagner, and our own: That is, parent sperm-cells in which are formed daughter-cells; in each of these last there is formed a spermatic particle. But Meissner is undecided if this formation occurs through a metamorphosis of the nucleus of the daughter cell. Our own observations have led us fully to think that this nucleus is thus metamorphosed, as we have expressed in a former review.*

The development of the egg is quite note-worthy, as it shows what we have before never clearly understood, viz: how botryoidal ovular masses are formed, and moreover carries out the beautiful analogy existing even to minute details, between the func-

* Am. Jour. of Science and Art, Nov., 1853., p. 393.

tions of the parent sperm-cell and the ovular cell. An ovular or egg-cell from the ovary is seen ; it increases in size and its nucleus segments, several nuclei being formed. These nuclei approach the surface of this which we will now call the parent egg-cell ; deverticula are given off from the cell-wall by a protrusion containing each a nucleus. These protrusions become constricted and at last appear as little, or daughter-cells, on the surface of the parent-cell. They now increase at the expense of this last, become pedunculated, and finally appear as larger pedunculated cells attached around a common, insignificant centre. These are the ova and form groups of variable number—Meissner having observed even twenty, though there are generally less. Thus formed, their peduncles break off and they pass from the ovary proper into the other sections of the genital tube.

There is one other point taken up in this connection by Meissner, and to which we briefly allude as it has been a subject of discussion on a former page.* We refer to the wonderful *Micropyle* of Keber, whereby it is alleged that the spermatic particles penetrate the interior of the egg and impregnate it. Meissner has seen nothing to justify the view that such a structure exists in the eggs of *Mermis*, excepting the remains of the peduncle above mentioned, and this he is not sure of being hollow. Moreover even if it were hollow, it appears to us wholly different from the special structure insisted upon by Keber.

As to the embryonic development of *Mermis*, our author found nothing essentially different from what had been described by previous observers upon this order, (Grube, Leidy, &c ) There appears to occur no proper metamorphosis, and therefore the newly hatched embryos more or less closely resemble in form, &c., the adults.

In conclusion, we repeat what we said in the beginning, that this memoir is one of the most excellent of its kind we have ever seen, and the care, patience and fidelity displayed therein will ensure attention towards its author as one from whom much may be expected.—*Am. Journal of Science and Art*, *July*, 1854.

---

* See a review on the doctrines of impregnation, in the Am. Journal, of Science and Art, Nov. No., 1853, p. 393.

